# Pushing the Boundaries of Biomarker Discovery in Phenylketonuria: Metabolomic Profiling Reveals Novel Biomarkers and Their Associations with Phenylalanine

**DOI:** 10.3390/molecules31122000

**Published:** 2026-06-08

**Authors:** Reem AlMalki, Shereen M. Aleidi, Maha Al Mogren, Shaima Almohsen, Khalid M. Sumaily, Ahmed Alfares, Anas M. Abdel Rahman

**Affiliations:** 1 Metabolomics Section, Precision Medicine Laboratory Department, Genomics Medicine Center of Excellence, King Faisal Specialist Hospital and Research Center, Riyadh 11211, Saudi Arabia; mmogren@kfshrc.edu.sa (M.A.M.); shalmohsen@kfshrc.edu.sa (S.A.); aalfares@kfshrc.edu.sa (A.A.); 2 College of Pharmacy, University of Sharjah, Sharjah 27272, United Arab Emirates; s.aleidi@ju.edu.jo; 3 Department of Biopharmaceutics and Clinical Pharmacy, School of Pharmacy, The University of Jordan, Amman 11942, Jordan; 4 Clinical Biochemistry Unit, Pathology Department, College of Medicine, King Saud University, Riyadh 11461, Saudi Arabia; ksumaily@ksu.edu.sa; 5 Department of Neuroscience and Renal, Bristol Medical School, Dorothy Hodgkin Building, Whitson Street, Bristol BS1 3NY, UK

**Keywords:** phenylketonuria, next-generation metabolic screening (NGMS), untargeted metabolomics, biomarker, phenylalanine

## Abstract

Background: Phenylketonuria (PKU) is a rare genetic disorder caused by mutations in the *phenylalanine hydroxylase (PAH)* gene, resulting in a deficiency of the enzyme responsible for metabolizing phenylalanine (Phe) and its accumulation. PKU can be identified through newborn screening (NBS) or genetic sequencing; however, both approaches have limitations, including high false-discovery rates and variants of uncertain significance (VUS). This study aims to identify a PKU metabolomic profile using unique biomarkers to enhance early diagnosis and improve treatment outcomes. Methods: Dried blood spot (DBS) samples from 65 patients diagnosed with PKU and matched healthy controls were collected through the NBS program. An untargeted metabolomics analysis was conducted using liquid chromatography-high-resolution mass spectrometry (LC-HRMS) to profile metabolites and investigate altered metabolic pathways in patients with PKU. Results: A total of 418 significantly dysregulated metabolites were identified in PKU patients. Among them, 90 metabolites were identified as endogenous human metabolites. The most significantly affected pathways were those related to the metabolism of aromatic amino acids and polysaccharides. Moreover, lipid metabolic pathways were dysregulated, including those involved in fatty acid and phospholipid biosynthesis. In addition to phenylalanine (AUC = 0.994), 1,11-Undecanedicarboxylic acid (UDCA) (AUC = 0.969) was significantly elevated in patients with PKU, suggesting it is a promising potential biomarker for PKU. Conclusions: Untargeted metabolomics revealed distinct metabolic alterations in patients with PKU, providing insights into disease pathophysiology. The identification of UDCA as a consistently elevated metabolite supports its potential utility as a supplementary biomarker for PKU diagnosis and monitoring. Further validation in larger cohorts, using a targeted metabolomics approach, is warranted.

## 1. Introduction

Phenylketonuria (PKU) is an inherited metabolic disorder caused by mutations in the PAH gene, which encodes the enzyme phenylalanine hydroxylase. This enzyme catalyzes the conversion of phenylalanine (Phe) to tyrosine (Tyr). This enzyme catalyses the conversion of phenylalanine (Phe) to tyrosine (Tyr) and requires tetrahydrobiopterin (BH4) as an essential cofactor for the hydroxylation of Phe [1,2]. Deficiency or mutations in PAH or in the enzyme that produces (BH4) lead to hyperphenylalaninemia or PKU [1]. Mild PAH deficiencies result in modest elevations of blood Phe, typically below 360 µmol/L, and generally do not require therapeutic intervention. In contrast, severe PAH deficiency leads to PKU, which is characterized by persistent elevations of blood Phe concentrations above 360 µmol/L [3,4]. If left untreated, these elevated Phe levels become neurotoxic, leading to impaired brain development, cognitive dysfunction, and irreversible intellectual disability [4]. Early diagnosis is crucial to start treatment promptly and prevent toxicity associated with high levels of Phe in the blood [5]. Treatment includes a Phe-restricted diet, which is challenging to maintain, and medications [6]. Importantly, approximately 30–50% of individuals with PAH deficiency exhibit significant reductions in blood Phe concentrations following treatment with sapropterin dihydrochloride, a synthetic form of BH4, underscoring the importance of BH4 responsiveness in personalised management of PKU [6].

Biochemically, PKU is characterized by elevated Phe concentrations and increased levels of secondary metabolites, such as phenylpyruvate, phenylacetate, and phenyllactate, reflecting altered Phe catabolism [7].

PKU is routinely identified through newborn screening (NBS) using tandem mass spectrometry (TMS) on dried blood spots (DBS) collected from newborns [8]. Elevated Phe levels, often assessed along with Phe-to-Tyr ratios, prompt further diagnostic evaluation. Confirmatory testing usually combines molecular genetic analysis with additional metabolic assessments to confirm the diagnosis and determine disease severity. Genetic testing for PKU typically employs high-throughput sequencing methods, such as whole-exome sequencing (WES) or targeted next-generation sequencing (NGS), to detect pathogenic mutations in the *PAH* gene [9].

Despite these advances, significant challenges remain in PKU screening and diagnosis [8]. False-positive results can occur where newborns are mistakenly identified as having PKU despite not having the disorder, leading to unnecessary follow-up testing and parental anxiety [10].

The reported positive predictive value (PPV) of approximately 13% in neonatal PKU screening, as described by Zhu et al. (2020) [11], underscores the complexity of the disorder’s pathophysiology. These findings suggest that traditional biomarkers do not fully capture the metabolic complexity of PKU, as transient neonatal factors or alternative metabolic states may influence Phe elevations. Conversely, false-negative results may be missed due to metabolic variability, delayed accumulation of Phe, or milder phenotypes. In addition, genetic testing frequently identifies variants of uncertain significance (VUS), complicating diagnostic interpretation and clinical decision-making. Functional analyses are often necessary to interpret these variants and determine their relevance to the clinical phenotype [12].

To address these limitations, recent research has focused on integrating untargeted metabolomics into inborn errors of metabolism (IEMs) screening and diagnostic workflows [13]. Although elevated Phe concentrations and Phe-to-Tyr ratios remain the gold-standard biomarkers for PKU diagnosis and clinical monitoring, these conventional markers do not fully capture the broader metabolic disturbances associated with the disorder. Untargeted metabolomics enables comprehensive profiling of metabolic perturbations beyond classical amino acid markers, revealing novel biochemical signatures and dysregulated pathways associated with PKU and other IEMs [14]. Such metabolic signatures may provide complementary information regarding disease heterogeneity, metabolic control, neurological involvement, and genotype–phenotype relationships. In addition, this approach has the potential to improve diagnostic sensitivity and specificity, reduce false-positive screening results, and enhance the interpretation of variants of uncertain significance (VUS), particularly when integrated with genomic data.

This study aims to perform an untargeted metabolomics analysis of DBS samples from individuals diagnosed with PKU to identify novel metabolic biomarkers and altered pathways that could improve early disease detection. Rather than replacing established biomarkers such as Phe and Tyr/Phe ratios, the goal of this study was to investigate whether broader metabolomic profiling could provide complementary insights into PKU pathophysiology and improve biochemical characterization of the disease. Using this approach, the study would enhance diagnostic accuracy and address challenges such as false-positive newborn screening results and phenotypic variability, thereby supporting more accurate interpretation of VUS in genetic testing. Ultimately, the identification of robust metabolic signatures may enable more precise diagnosis, optimized clinical management, and personalized therapeutic strategies for individuals with PKU.

## 2. Results

### 2.1. The Demographic and Clinical Characteristics of the Study Groups

The demographic and clinical characteristics of the phenylketonuria (PKU) group (*n* = 65) and healthy controls (*n* = 65) are shown in Table 1. Both groups were matched by age and gender. They had an equal percentage of females (58.5%). Additionally, the mean age of participants did not differ significantly between the PKU group (13.18 ± 9.9 years) and the control group (13.6 ± 13 years). However, the mean Phe level was significantly higher in the PKU group (743.4 ± 320.31 µmol/L) than in the control group (38.0 ± 10.2 µmol/L; *p* < 0.05). Likewise, the Phe-to-Tyr concentration ratio was substantially higher in PKU patients (17.3 ± 10.5) compared to healthy individuals (1.7 ± 0.4; *p* < 0.05).

### 2.2. Metabolomics Profiling of PKU Patients Compared to Controls

**Figure 1:**
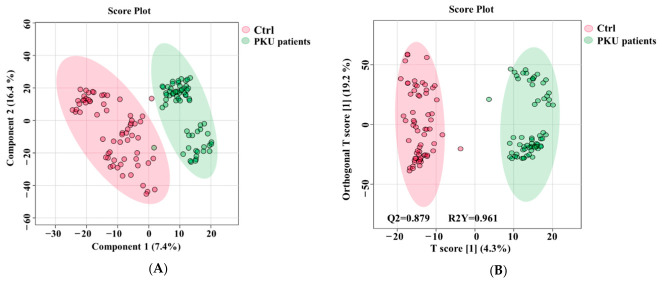
(**A**) Partial least squares discriminant analysis (PLS-DA) displaying separation between groups. (**B**) The OPLS-DA model shows evident separation between PKU patients and Ctrl. The robustness of the created models was evaluated using the model’s fitness (R2Y = 0.961) and predictive ability (Q2 = 0.879) on a larger dataset (*n* = 100).

The metabolomics profiling of PKU patients was performed using liquid chromatography-high resolution mass spectrometry (LC-HRMS) (Figure S1). 16,872 mass ion features were detected in both positive (*n* = 10,925) and negative (*n* = 5947) ionization modes. After applying a frequency-based filter with an 80% cutoff across all samples, 8374 features remained for further analysis. Partial least squares discriminant analysis (PLS-DA) displays clustering between the PKU and control groups (Figure 1A), and Cross-validation analysis demonstrates stable predictive performance across latent components, with the selected model showing an optimal balance between predictive ability (Q^2^) and model complexity (Figure S2). Furthermore, the supervised OPLS-DA analysis showed clear separation and grouping between PKU and control subjects, with the robustness of the created models evaluated by the fitness of the model (R2Y = 0.961) and predictive ability (Q2 = 0.879) values in a larger dataset (*n* = 100) using permutation analysis (Figure 1B). These findings indicate a significant difference in the metabolic expression of the study groups.

**Figure 2:**
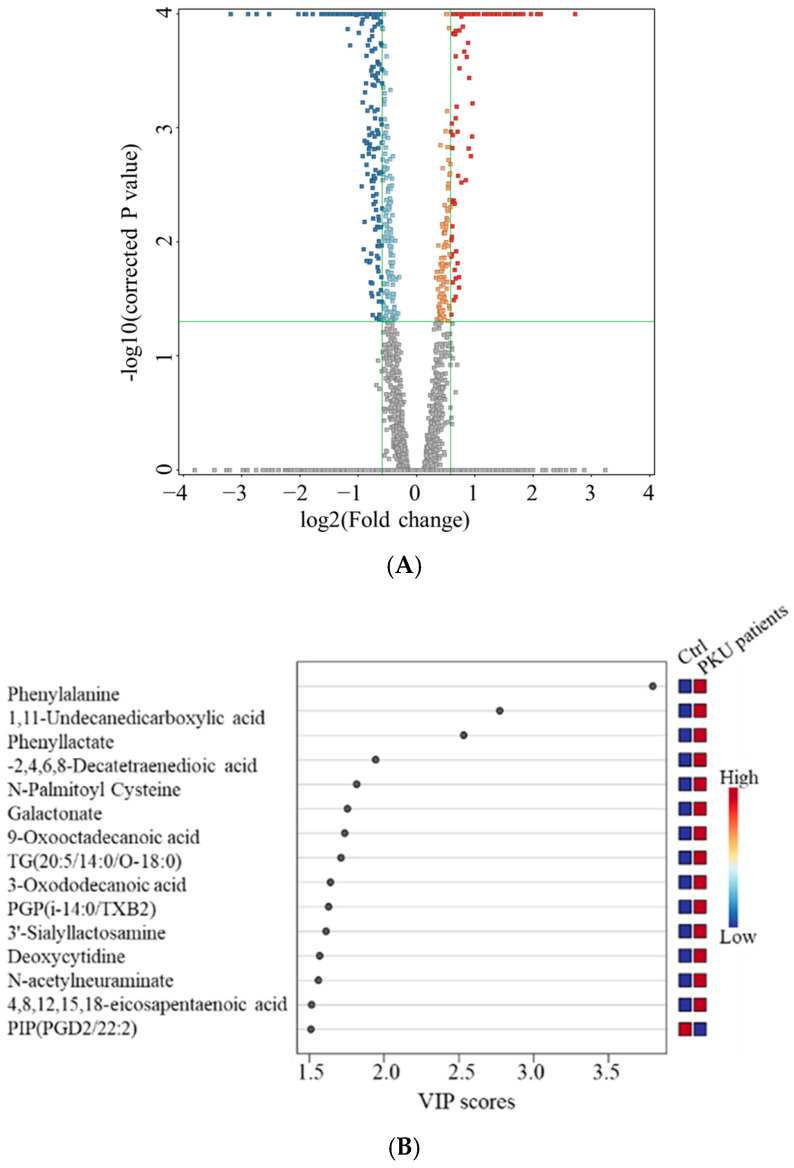
(**A**) A volcano plot shows significantly dysregulated metabolites in PKU patients compared to controls: 418 metabolites were significantly dysregulated, with 115 up (red) and 303 down (blue). (**B**) VIP score showing the top 15 significantly endogenous metabolites.

A binary comparison between PKU patients and healthy controls using volcano plot analysis (Moderated *t*-test FDR-*p* < 0.05, FC 1.5) revealed that 418 metabolites were significantly dysregulated (Figure 2A). Among them, 115 and 303 were up- and down-regulated, respectively, in PKU patients compared to controls. Out of 418, only 207 were annotated, where, after excluding the exogenous molecules (i.e., drugs, drug metabolites, environmental exposures, etc.), 90 metabolites remained as human endogenous metabolites. VIP scores represented the top 15 significantly dysregulated metabolites, as shown in Figure 2B. As expected, Phe, phenyllactate, and tyrosine were among the biomarkers reflecting known biochemical pathways of PKU, with Phe as the principal diagnostic biomarker (Table S1). Among these, the annotation of the 9 metabolites was verified using our in-house library built based on reference standard materials (Table S2).

### 2.3. Pathway and Enrichment Analyses of the Significantly Dysregulated Metabolites in PKU Patients Compared to Healthy Controls

**Figure 3:**
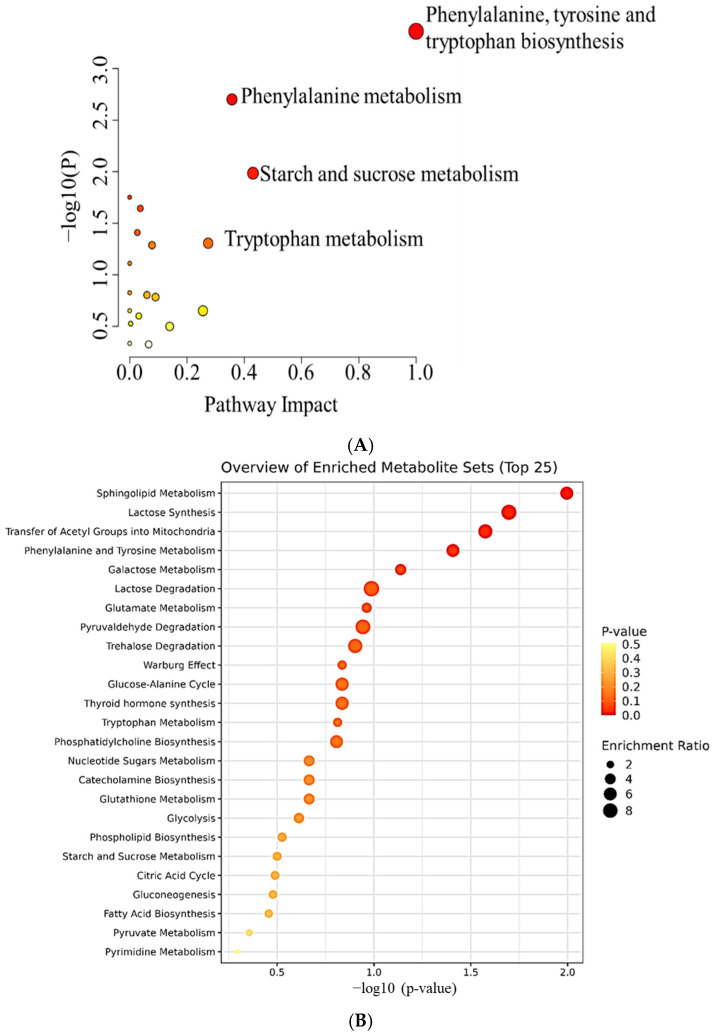

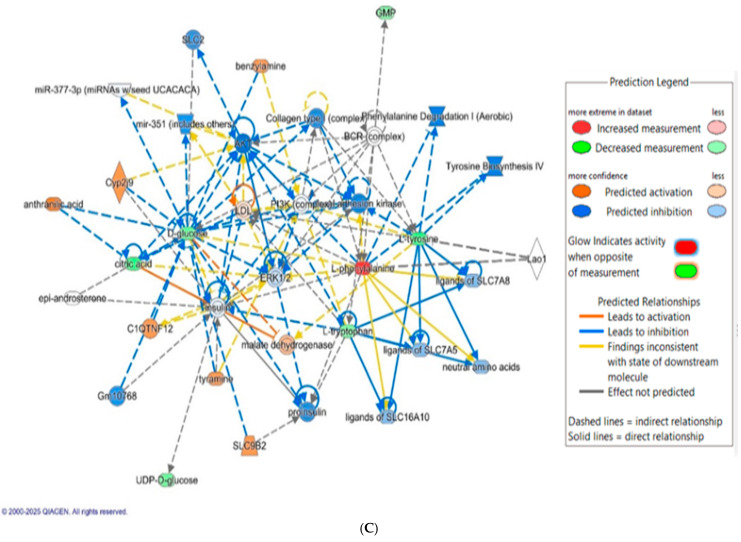
Pathway and enrichment analysis of the significantly dysregulated metabolites in PKU patients compared to healthy controls. (**A**) Pathway analysis of 90 significantly dysregulated metabolites in PKU patients compared to healthy controls. (**B**) Enrichment analysis showing the top 25 dysregulated metabolites in the pathway. The *y*-axis lists metabolite pathways, and the *x*-axis represents statistical significance expressed as −log10 (*p*-value), with values further to the right indicating stronger significance. Each bubble corresponds to a metabolite set. Bubble size reflects the enrichment ratio (larger bubbles indicate greater enrichment), and bubble colour represents the *p*-value, with darker red shades indicating higher statistical significance (lower *p*-values). (**C**) IPA network analysis represents predicted molecular interactions and activity states. Nodes represent molecules and edges represent curated or predicted relationships between them.

A pathway analysis was performed on 90 significantly dysregulated endogenous metabolites, highlighting the most affected pathways in PKU. The primary pathways involved amino acid and polysaccharide metabolism, such as Phe, tyrosine, and tryptophan biosynthesis, as well as Phe, starch, sucrose, and tryptophan metabolism (Figure 3A). Additionally, enrichment analysis of these dysregulated metabolites identified the top 25 most enriched sets, with prominent pathways including sphingolipid metabolism, lactose synthesis, and mitochondrial acetyl group transfer (Figure 3B). Conversely, pathways like pyrimidine and pyruvate metabolism showed lower enrichment and statistical significance (Figure 3B). Ingenuity pathway analysis (IPA) further revealed increased Phe levels and reduced levels of tyrosine, tryptophan, glucose, UDP-glucose, and citric acid cycle metabolites (Figure 3C).

### 2.4. Biomarker Evaluation

**Figure 4:**
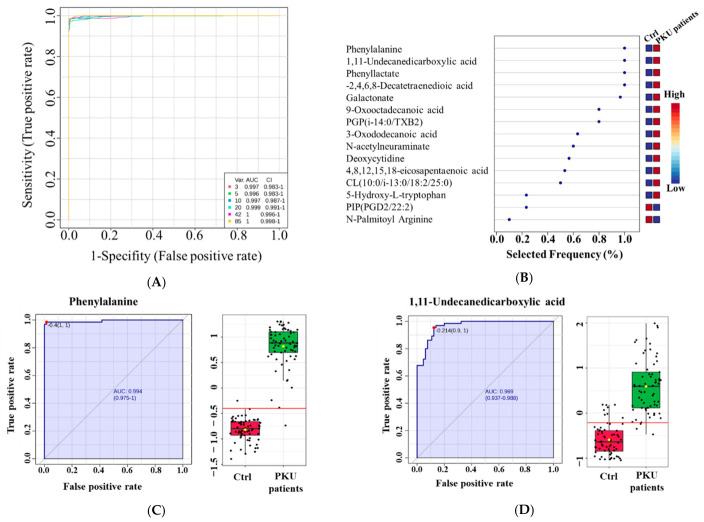
Biomarker evaluation between PKU patients and controls. (**A**) Receiver operating characteristic (ROC) curve comparing PKU patients with controls. It was generated by the OPLS-DA model, with AUC values calculated using combinations of 3, 5, 10, 20, 42, and 85 metabolites. (**B**) A frequency plot shows the significantly dysregulated endogenous metabolites in patients with PKU compared to controls. ROC curves for individual biomarkers: (**C**) Phenylalanine (AUC = 0.994) and (**D**) 1,11-Undecanedicarboxylic acid (AUC = 0.969) were upregulated in PKU patients compared to Ctrl.

A multivariate exploratory receiver operating characteristic (ROC) analysis was generated based on the identified significantly dysregulated metabolites (*n* = 90) between PKU and controls. OPLS-DA was used for classification and feature ranking. ROC curves of the top-ranked metabolites illustrated that the area under the curve (AUC) ranged from 0.997 to 1, with confidence intervals (CI) of 0.983–1 and 0.998–1 (Figure 4A). The significant features of the positively identified metabolites are presented in Figure 4B, which shows 15 metabolites with the highest VIP scores in the OPLS-DA model, based on their levels in PKU patients. Levels of 13 out of these 15 metabolites were upregulated in PKU patients compared to healthy controls. Among these upregulated metabolites, the top two, Phe and 1,11-Undecanedicarboxylic acid, were represented in the ROC curve with AUC values of 0.994 and 0.969, respectively (Figure 4C,D). In addition, chemical structures and ratio-based biomarkers (Figures S3 and S4) demonstrated enhanced diagnostic performance. Phenylalanine-based ratios exhibited strong discriminatory power with near-perfect accuracy, confirming the central role of Phe in PKU pathology. Notably, metabolite ratios such as Tyr/Phe, mannitol 1-phosphate/Phe, and DL-glutamate/Phe achieved perfect classification performance, highlighting the improved sensitivity of ratio-based markers over individual metabolites.

## 3. Discussion

Phenylketonuria (PKU) is a rare, autosomal recessive metabolic disorder affecting the metabolism of aromatic amino acids. It is characterized by the accumulation of Phe in the blood and brain tissue and is associated with an increased risk of neurotoxicity and brain damage [15]. Neonatal screening programs are crucial, as they facilitate early diagnosis and timely management through dietary Phe restriction and medical treatments [9,16]. This can significantly reduce the risk of irreversible neurodevelopmental damage [9,16].

However, challenges are associated with PKU management, particularly in distinguishing between phenotypic variations and predicting treatment responsiveness. This led to growing interest in employing advanced analytics, such as metabolomics, a comprehensive, high-throughput analysis using mass spectrometry. Metabolomics research in PKU is relevant, as it enables the identification of novel biomarkers that offer thorough insights into the biochemical changes associated with PKU [17,18]. Moreover, identifying new biomarkers for PKU through metabolomics analysis would significantly enhance the monitoring of disease progression and patients’ response to therapeutic approaches.

In this study, an untargeted MS-based metabolomics approach was used to analyze dysregulated metabolites and examine altered biochemical pathways in patients with PKU. Multivariate statistical analysis, including PLS-DA and OPLS-DA, demonstrated clear separation and clustering between the PKU and control groups. This indicates a significant difference in metabolite expression between the two groups. Volcano plot analysis identified significant dysregulation in 418 metabolites, with 115 upregulated and 303 downregulated in PKU compared to controls. After metabolite annotation, 117 metabolites were classified as exogenous, including drugs, drug metabolites, and environmental exposures. This underscores the influence of the exposome, representing human environmental exposures throughout life, and gene-environment interactions in PKU outcomes [19]. Recently, increasing attention has been paid to the role of exposure in the etiology and development of diseases. In the case of PKU, identifying early-life exposures, especially during pregnancy and the neonatal period, presents a new and important challenge for advancing our understanding of this metabolic disorder. Further research is needed to explore the role of the exposome and its potential impact on PKU diagnosis, progression, and management.

After excluding exogenous metabolites, 90 endogenous metabolites were identified as significantly dysregulated in PKU patients. Pathway and enrichment analysis revealed that alterations in aromatic amino acid metabolism, specifically Phe, Tyr, and tryptophan, predominantly characterize the metabolic signature in PKU patients. This is consistent with the known amino acid dysregulation in patients with PKU. PKU is associated with mutations in the PAH gene, located on chromosome 12, leading to enzyme deficiency or insufficiency [4,20,21]. This enzyme mediates the rate-limiting step in the irreversible hydroxylation of Phe to form Tyr in a reaction that requires tetrahydrobiopterin (BH4) as a cofactor [9,20]. Tyr is a non-essential amino acid that is necessary for synthesizing melanin and acts as a precursor for several neurotransmitters, including dopamine, epinephrine, and norepinephrine [22]. In physiologically normal individuals, the Phe pool is derived primarily from two sources: dietary proteins and the catabolic turnover of endogenous polypeptides [23]. Approximately 25% of this free Phe pool is utilized for protein synthesis, while the majority (~75%) undergoes hydroxylation to Tyr via the PAH enzyme [23]. Reduced or absent activity of PAH would accumulate Phe (hyperphenylalaninemia) and consequently decrease the availability of Tyr, leading to tyrosine deficiency [4,24]. Moreover, defects in PAH would lead to the accumulation of Phe- related metabolites, such as phenyl ketones (e.g., phenylpyruvate and phenyllactate), as well as other Phe-metabolism products [4]. In our analysis, Phe and phenyllactate were among the upregulated metabolites, whereas Tyr was downregulated in PKU patients.

It has been indicated that accumulating Phe would interfere with myelin production in the central nervous system via impairing the synthesis of cholesterol and other brain lipids [25]. One of the suggested mechanisms underlying the lipid disturbances associated with high Phe levels is the inhibition of 3-hydroxy-3-methylglutaryl-CoA (HMG-CoA) reductase activity [9,25]. In line with this, our pathway and enrichment analyses revealed significant perturbations in lipid metabolism, particularly in sphingolipid and phosphatidylcholine pathways, suggesting that disturbances in membrane lipid homeostasis may represent an important component of PKU pathophysiology. This finding is biologically plausible given that myelin, which is essential for rapid nerve conduction and normal neurodevelopment, consists of approximately 70–85% lipids by dry weight, with cholesterol, sphingolipids, and phosphatidylcholine-derived phospholipids serving as major structural constituents [26]. Alterations in these lipid classes can compromise myelin formation and potentially contribute to the white matter abnormalities and neurocognitive deficits frequently observed in individuals with PKU. Experimental studies have demonstrated that elevated phenylalanine concentrations can directly interfere with myelin membrane organization and induce demyelination [27], whereas reviews of PKU neuropathophysiology consistently implicate altered lipid metabolism as a key contributor to hypomyelination and white matter injury [28,29]. The observed disturbances in sphingolipid and phosphatidylcholine metabolism in our cohort therefore suggest that PKU is associated not only with abnormalities in amino acid metabolism but also with broader alterations in structural and signaling lipids critical to central nervous system integrity. These findings highlight the potential value of lipidomic biomarkers for assessing disease severity, treatment response, and long-term neurodevelopmental outcomes.

In addition to Phe accumulation, elevated phenyllactate may also contribute to PKU pathophysiology. Phenyllactate, a secondary metabolite generated via alternative phenylalanine-catabolic pathways, has been associated with mitochondrial dysfunction, oxidative stress, and neurotoxicity in hyperphenylalaninemic states [28,30]. Increased phenyllactate levels may further exacerbate neuronal injury by disrupting cellular energy metabolism and redox homeostasis, thereby amplifying the neurological manifestations of PKU. Supporting this concept, our pathway and enrichment analyses identified significant perturbations in carbohydrate-related pathways, including lactose, starch, and sucrose metabolism, as well as galactose metabolism. These findings likely reflect broader disturbances in intermediary carbohydrate and energy metabolism rather than primary defects in the digestion or utilization of specific dietary sugars. In our Ingenuity Pathway Analysis, reduced levels of glucose and UDP-glucose further support disruptions in glycogen synthesis, glycosylation, and energy homeostasis. Several mechanisms may contribute to these abnormalities in PKU. Recent metabolomics studies of patients with PKU have highlighted the metabolic disturbances associated with this disorder. For example, Blasco et al. employed multiplatform metabolomics to examine the metabolic signature of patients with PKU using plasma and urine samples [31]. The study corroborated the contribution of multiple biochemical pathways in PKU pathophysiology, specifically alterations in protein synthesis, disturbances in energy metabolism, and increased oxidative stress [31].

In addition, Wegberg et al. highlighted the clinical relevance of 11 metabolites previously identified using targeted metabolomics [7]. Among these metabolites investigated, Phe and N-lactoyl-phenylalanine were significantly associated with attention-deficit/hyperactivity disorder (ADHD) symptoms in patients with PKU [7]. Notably, N-lactoyl-phenylalanine demonstrated stronger associations with working memory performance and mental health outcomes than Phe [7]. The findings of our study, along with those reported by Wegberg, underscore the involvement of lactose metabolism in PKU, thereby emphasizing the potential clinical relevance of incorporating lactose restriction into dietary management strategies for patients with PKU. Within this scope of investigation, a separate study by Schoen and Singh examined the short-term metabolic changes in the plasma of females with PKU following nutritional interventions [32]. Compared with healthy controls, females with PKU exhibited elevated baseline levels of Phe catabolites, ketone bodies, and carnitine- and glycine-conjugated fatty acids. In contrast, levels of fatty acylcholine metabolites were significantly reduced after specific diet restriction [32]. The study identified alterations in metabolic pathways beyond Phe metabolism, outlining the depth of metabolic changes in response to dietary management [32]. In this context, our study highlights the involvement of various complex biochemical pathways, including Phe and amino acid metabolism, the biosynthesis of fatty acids, phospholipids, and phosphatidylcholine, and the transfer of the acetyl group to mitochondria.

Notably, our analysis showed that 1,11-Undecanedicarboxylic acid (UDCA) (HMDB0000623), a dicarboxylic acid, was significantly elevated in patients with PKU, with an AUC of 0.969, indicating strong potential as a promising biomarker for PKU. This metabolite belongs to the class of organic compounds known as long-chain fatty acids with an aliphatic tail containing two terminal carboxylic acid groups and a nine-carbon alkyl chain in between [33]. This metabolite is typically produced in the body via the omega-oxidation of fatty acids [34,35]. Elevated levels of 1,11-Undecanedicarboxylic acid (UDCA) could reflect dysregulation of fatty acid oxidation or peroxisomal dysfunction. The consistently high levels observed in our cohort underscore the potential relevance of this metabolite within broader metabolic networks affected in PKU. These findings suggest the need for further investigation into UDCA’s potential as a biomarker of metabolic dysregulation in PKU patients.

Several potential confounding variables known to influence metabolomic profiles in PKU, including dietary intake, treatment adherence, metabolic control, medication use, and nutritional supplementation, could not be fully controlled due to the retrospective study design and limited availability of detailed clinical information. Consequently, some of the observed metabolic alterations may reflect differences in dietary management or therapeutic interventions in addition to disease-specific biochemical disturbances. In addition, external validation using an independent cohort of well-characterized PKU DBS samples is needed. Future prospective studies integrating larger patient populations, standardized dietary assessment, targeted metabolomics validation, longitudinal clinical monitoring, detailed treatment and medication data, and functional investigations will be important to confirm the biological significance and clinical applicability of the identified metabolic signatures.

## 4. Materials and Methods

### 4.1. Patient Inclusion and DBS Collection

In this study, DBS cards were sourced from the metabolomics section at the Genomic Medicine Center of Excellence, KFSHRC in Riyadh. The study included DBS cards from 65 patients with PKU and 65 age- and gender-matched healthy controls. Patients were included if they had a biochemical diagnosis and confirmation through phenotypic and/or genetic assessments. Participants who did not meet these criteria were excluded.

### 4.2. Ethical Consideration

The Institutional Review Boards at King Faisal Specialist Hospital and Research Centre (KFSHRC) in Riyadh, Saudi Arabia (RAC# 2160 027) reviewed and approved this study and its related procedures. In agreement with KFSHRC’s institutional and national legislation. Patient samples and information were obtained from leftover materials and the requisition form from routine clinical services; patient consent was not required for their use.

### 4.3. Sample Preparation

Metabolites were extracted from the DBS using a standard procedure [36]. One punch of DBS with a diameter of 3.2 mm was immersed in 300 μL of (distilled water: methanol, acetonitrile) (dH_2_O:MeOH:ACN) at a ratio of 20:40:40% as the extraction solvent. The samples were vortexed in a ThermoMixer (Eppendorf, Hamburg, Germany) at 600 rpm and 25 °C for 2 h. Subsequently, the samples were centrifuged at 16,000 rpm at 4 °C for 10 min. The supernatants were transferred and evaporated in a Speed-Vac (Christ, Osterode am Harz, Germany). The dried samples were reconstituted with 100 μL of mobile phase A:B (1:1), where A is 0.1% formic acid in dH_2_O and B is 0.1% formic acid in 50% ACN:MeOH.

### 4.4. LC-MS Metabolomics

Metabolic fingerprints were analyzed using the Waters Acquity UPLC system coupled to an Xevo G2-S QTOF mass spectrometer equipped with an electrospray ionization (ESI) source (Waters Corporation, Milford, MA, USA), as previously reported [36]. The extracted metabolites were chromatographed with an ACQUITY UPLC system using a (HSS T 31.8 µm, 2.1 × 100 mm) column (Waters Ltd., Elstree, UK). The mobile phase consisted of 0.1% formic acid in dH_2_O as solvent A, and B was 0.1% formic acid in a 1:1 *v*/*v* mixture of ACN and MeOH. A gradient elution schedule was applied as follows: 0–16 min, 5–95% A; 16–19 min, 5% A; 19–20 min, 5–95% A; and 20–22 min, 5–95% A, at a flow rate of 300 µL/min. MS spectra were acquired in positive- and negative-ion electrospray ionization (ESI+) and (ESI−) modes. MS conditions included a source temperature of 150 °C, a desolvation temperature of 500 °C, a capillary voltage of 3.20 kV (ESI+) or 3 kV (ESI−), a cone voltage of 40 V, desolvation gas flow at 800.0 L/h, and cone gas flow at 50 L/h. The collision energies for low and high functions were set to off and 10 V to 50 V, respectively, in MSE mode. The mass spectrometer was calibrated using sodium formate over the mass range 100–1200 Da in both ionization modes. The lock mass compound, leucine-enkephalin (an external reference at *m*/*z* 556.2771 for ESI+ and 554.2615 for ESI−), was infused continuously, switching between the sample and the reference every 45 s for ESI+ and 60 s for ESI−. The scan time was 0.5 s, flow rate was 10 µL/min, cone voltage was 30 V, and collision energy was 4 V. Data were collected in continuum mode with MassLynx ™ V 4.1 workstation (Waters Inc., Milford, MA, USA). Quality control (QC) samples were prepared by collecting a single 3.2 mm punch from each study sample, pooling them for extraction, and introducing them into the instrument in a randomized order to validate the system’s stability. The acceptance criteria required that all QC samples be separated from other study groups, clustered together, and have a relative standard deviation (RSD) ≤ 30%.

### 4.5. Data and Statistical Analyses

The MS raw data were processed using a standard pipeline that began with alignment based on *m*/*z* value and the retention time of the ion signals, followed by peak picking and signal filtering based on peak quality, all performed with the Progenesis QI v.3.0 software from Waters (Waters Technologies, Milford, MA, USA). Multivariate statistical analysis was performed using MetaboAnalyst version 6.0 (McGill University, Montreal, QC, Canada) (http://www.metaboanalyst.ca, accessed on 10 March 2025) [37]. The datasets (compound names and raw abundances) were median-normalized, Pareto-scaled, and log-transformed to ensure normality. The normalized datasets were used to generate partial least squares discriminant analysis (PLS-DA) and orthogonal partial least squares discriminant analysis (OPLS-DA) models. The OPLS-DA models were evaluated using the model fitness (R2Y) and predictive ability (Q2) metrics. Model validity was assessed by permutation testing (*n* = 100) [38]. To reduce the risk of overfitting and assess model robustness, 5-fold cross-validation was performed across multiple latent components, and the optimal number of components was selected based on the best balance between predictive performance and model complexity. Cross-validation performance metrics, including classification accuracy, R^2^, and Q^2^ values, are presented in Figure S2. Univariate analysis was performed using Mass Profiler Professional (MPP) v.15.0 software (Agilent, Santa Clara, CA, USA). A volcano plot was used to identify significantly altered mass features based on a Moderated *t*-test, with a cut-off of FDR-adjusted *p* < 0.05 and a fold change of 1.5 compared to controls.

### 4.6. Metabolites Identification

The significant features in each dataset were selected and tagged in Progenesis QI software for annotation. The chemical structures of metabolites were identified by acquiring their accurate precursor masses. Theoretical MS/MS fragmentation tolerance values were set to a 5 ppm mass window for the Human Metabolome Database (HMDB) [33] and 5 ppm for METLIN MS/MS (https://metlin.scripps.edu/landing_page.php?pgcontent=mainPage) (accessed on 1 April 2025)) using fragmentations filtered by in silico or empirical, KEGG, Lipid Map, and Lipid Blast. Metabolites were confirmed using in-house library reference standards and classified as MSI level 1 identifications, while the remaining metabolites were considered putatively annotated (MSI level 2) according to Metabolomics Standards Initiative criteria. Exogenous compounds, such as drugs, food additives, and environmental pollutants, were manually excluded from the final list.

### 4.7. Pathway, Biomarkers, Enrichment, and Network Analysis

Pathway analysis, biomarkers linked with PKU disorder, and receiver operating characteristic ROC curves were generated in the MetaboAnalyst (v.6.0) by Monte Carlo cross-validation (MCCV) with balanced sub-sampling. During each MCCV iteration, two-thirds (2/3) of the samples were designated for assessing feature importance. The top-ranked features, as determined by Partial Least Squares Discriminant Analysis (PLS-DA), were subsequently used to construct classification models. These models were then validated on the remaining one-third (1/3) of the samples. This procedure was repeated to assess each model’s performance and to compute its confidence interval. The PLS-DA algorithm was utilized for feature ranking with latent variables (LV) as 2. The value was considered only if the provided LV count was less than the number of features.

To determine whether dysregulation(s) in specific metabolite classes are associated with patients with PKU, metabolite set enrichment analysis (MSEA) was performed using chemical similarity (main class) based on the Global test in MetaboAnalyst v. 6, accessed on 11 May 2025 (McGill University, Montreal, QC, Canada; http://metaboanalyst.ca (accessed on 3 April 2025)). This analysis was based on annotated metabolites that were differentially dysregulated between PKU patients and controls, identified by volcano plot analysis (FDR-adjusted *p* < 0.1; FC cutoff 2).

Pathway and network analyses were performed using Ingenuity Pathway Analysis (IPA, QIAGEN, Redwood City, CA, USA).

Identified metabolites were uploaded into the IPA platform and mapped to corresponding molecular entities within the Ingenuity Knowledge Base. Core analyses were conducted to identify significantly enriched canonical pathways, molecular interaction networks, and potential upstream regulators associated with the metabolomic dataset. Statistical significance of pathway enrichment was determined using right-tailed Fisher’s exact test as implemented in IPA. This integrative approach enabled the interpretation of metabolite alterations within the context of curated biological interactions and experimentally validated molecular mechanisms.

## 5. Conclusions

This study offers valuable insights into the metabolic changes associated with PKU by identifying significant differences in metabolic profiles between patients with PKU and healthy controls. The study identified 90 human endogenous metabolites significantly dysregulated in patients with PKU. Moreover, it highlights the dysregulation of key metabolic pathways involved in amino acid and polysaccharide metabolism. These include phenylalanine, tyrosine, and tryptophan biosynthesis; phenylalanine metabolism; starch and sucrose metabolism; and fatty acid and phospholipid biosynthesis, indicating specific pathways that could be targeted for early screening and intervention. This research contributes significantly to the growing body of knowledge on the biochemical changes associated with PKU, offering a promising avenue for early prediction and diagnosis, as well as more effective management of the common metabolic disorder.

## Figures and Tables

**Table 1:** Demographic data of PKU patients and healthy controls.

Demographics and Clinical Characteristics	PKU (*n* = 65)	Controls (*n* = 65)	*p*-Value
	Mean ± STD	Mean ± STD	
Age (Years)	13.18 ± 9.9	13.6 ± 13	0.84
Female (%)	58.5	58.5	NA
Phe level (cutoff: <180)	743.4 ± 320.31	38.0 ± 10.2	3.0 × 10^−36^
phe/Tyr concentration ratio (cutoff: <3.1)	17.3 ± 10.5	1.7 ± 0.4	3.4 × 10^−22^

Data presented in Mean ± Standard Deviation; NA, not applicable.

## Data Availability

Data available upon request.

## Supplementary Materials

The following supporting information can be downloaded at: https://www.mdpi.com/article/10.3390/molecules31122000/s1, Figure S1: Representative LC–MS chromatograms and extracted ion chromatograms obtained from DBS samples analyzed in this study. (A) Total ion chromatogram (TIC) acquired in positive ionization mode. (B) Total ion chromatogram (TIC) acquired in negative ionization mode. (C) Extracted ion chromatogram of L-phenylalanine detected in negative ionization mode (retention time: 2.99 min; *m*/*z* 164.0714). (D) Extracted ion chromatogram of 1,11-undecanedicarboxylic acid detected in negative ionization mode (retention time: 12.22 min; *m*/*z* 243.1613). These chromatograms demonstrate the chromatographic separation and detection of representative metabolites identified in the untargeted metabolomics analysis; Figure S2: Cross-validated performance of the PLS model as a function of the number of latent components (1–5). Model performance was evaluated using 5-fold cross-validation. Bars represent classification accuracy (blue), goodness of fit (R^2^, pink), and predictive ability (Q^2^, light teal). Accuracy and R^2^ increase with additional components, reflecting improved model fit, while Q^2^ indicates predictive performance estimated from cross-validation. The red asterisk denotes the selected optimal number of components, providing the best balance between predictive performance and model complexity; Figure S3: Chemical structures of major metabolites discussed in this study. Aromatic amino acids, including (A): L-tryptophan, (B): L-phenylalanine, and (C): L-tyrosine. (D): Dicarboxylic acids, including 1,11-undecanedicarboxylic acid. Figure S4: Biomarker evaluation between PKU patients and controls. (A) Receiver operating characteristic (ROC) curve comparing PKU patients with controls. It was generated by the OPLS-DA model, with AUC values calculated from the combination of 5, 10, 15, 25, 50 and 100 metabolites. (B) A frequency plot shows the significantly dysregulated endogenous metabolites in patients with PKU compared to controls. ROC curves for individual biomarkers: (C) Phen (AUC = 0.993) was upregulated in PKU patients compared with controls. (D) Tyr/Phe (AUC = 1), (E): Mannitol 1-phosphate/Phe (AUC = 1), and (F) DL-Glutamate/Phe (AUC = 1) were downregulated in PKU patients compared to Ctrl; Table S1: Endogenous metabolites. (Moderated *t*-test, FDR *p*-value < 0.05, Fold change 1.5); Table S2: Endogenous metabolites identified based on the in-house library. (Moderated *t*-test, FDR *p*-value < 0.05, Fold change 1.5).
