# Supplementary material for: Pushing the Boundaries of Biomarker Discovery in Phenylketonuria: Metabolomic Profiling Reveals Novel Biomarkers and Their Associations with Phenylalanine
Source: Molecules. 2026 Jun 8;31(12):2000. doi: 10.3390/molecules31122000 (PMC13304883; doi:10.3390/molecules31122000)
Supplement: Supplementary file 1 [file molecules-31-02000-s001.zip › molecules-4312602-supplementary figures.pdf]

(A)

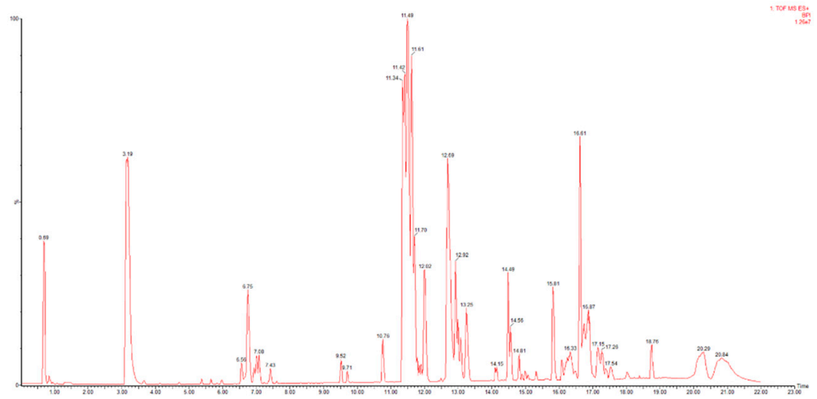

(B)

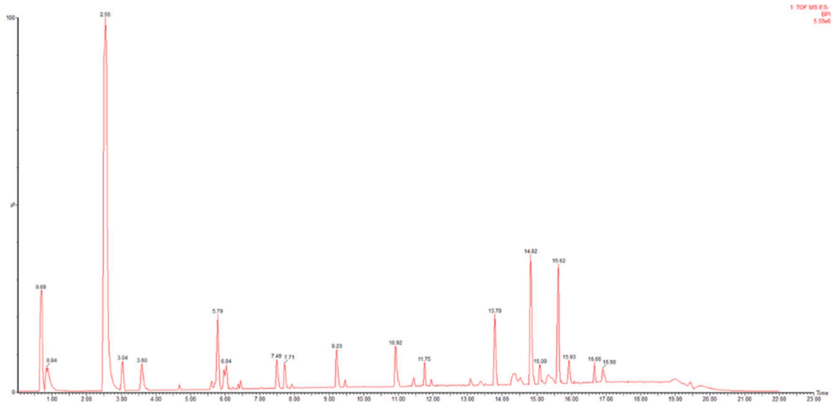

(C)

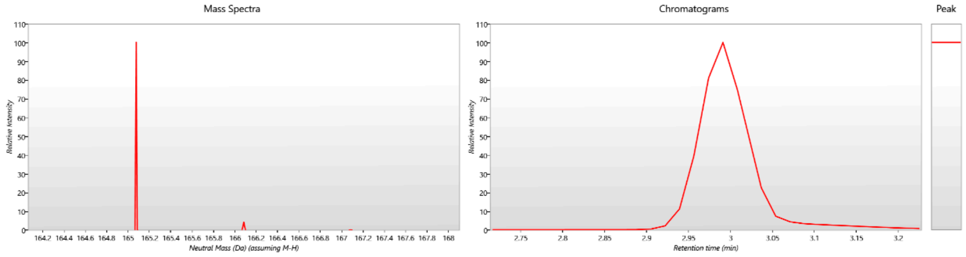
Mass Spectra
Relative Intensity
Neutral Mass (Da) (assuming M-H)
Chromatograms
Relative Intensity
Retention time (min)
Peak

(D)

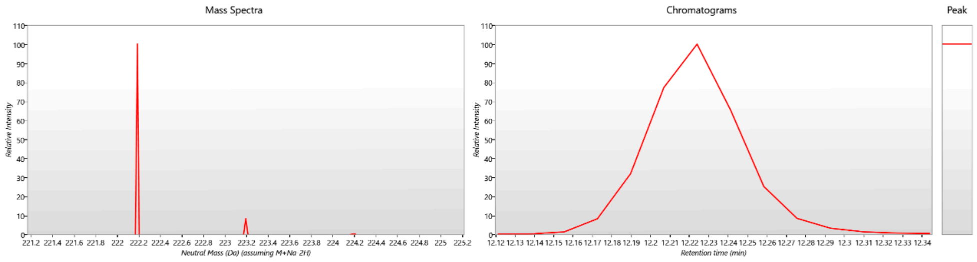

Figure S1. Representative LC–MS chromatograms and extracted ion chromatograms obtained from DBS samples analyzed in this study. (A) Total ion chromatogram (TIC) acquired in positive ionization mode. (B) Total ion chromatogram (TIC) acquired in negative ionization mode. (C) Extracted ion chromatogram of L-phenylalanine detected in negative ionization mode (retention time: 2.99 min; m/z 164.0714). (D) Extracted ion chromatogram of 1,11-undecanedicarboxylic acid detected in negative ionization mode (retention time: 12.22 min; m/z 243.1613). These chromatograms demonstrate the chromatographic separation and detection of representative metabolites identified in the untargeted metabolomics analysis.

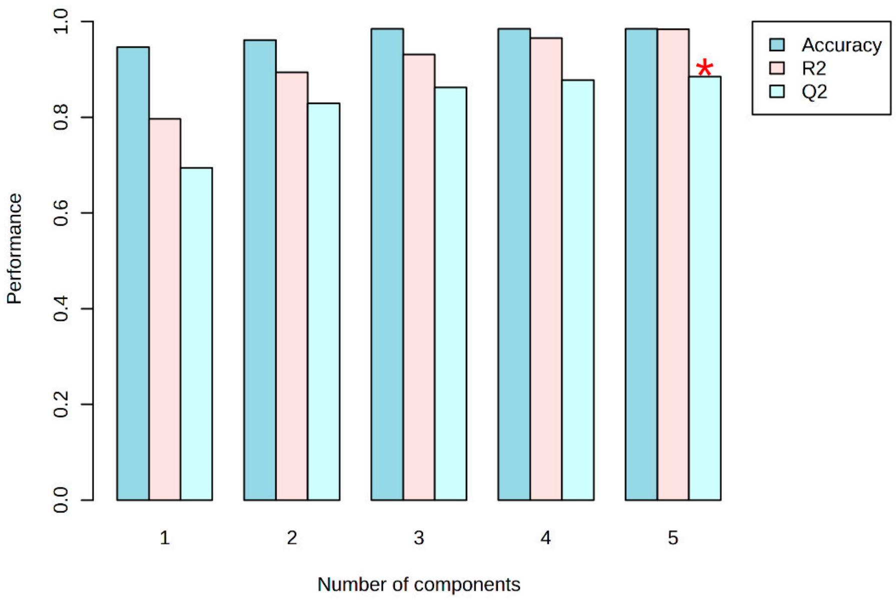

Figure S2. Cross-validated performance of the PLS model as a function of the number of latent components (1–5). Model performance was evaluated using 5-fold cross-validation. Bars represent classification accuracy (blue), goodness of fit (R^2, pink), and predictive ability (Q^2, light teal). Accuracy and R^2 increase with additional components, reflecting improved model fit, while Q^2 indicates predictive performance estimated from cross-validation. The red asterisk denotes the selected optimal number of components, providing the best balance between predictive performance and model complexity.

Classification: Restricted

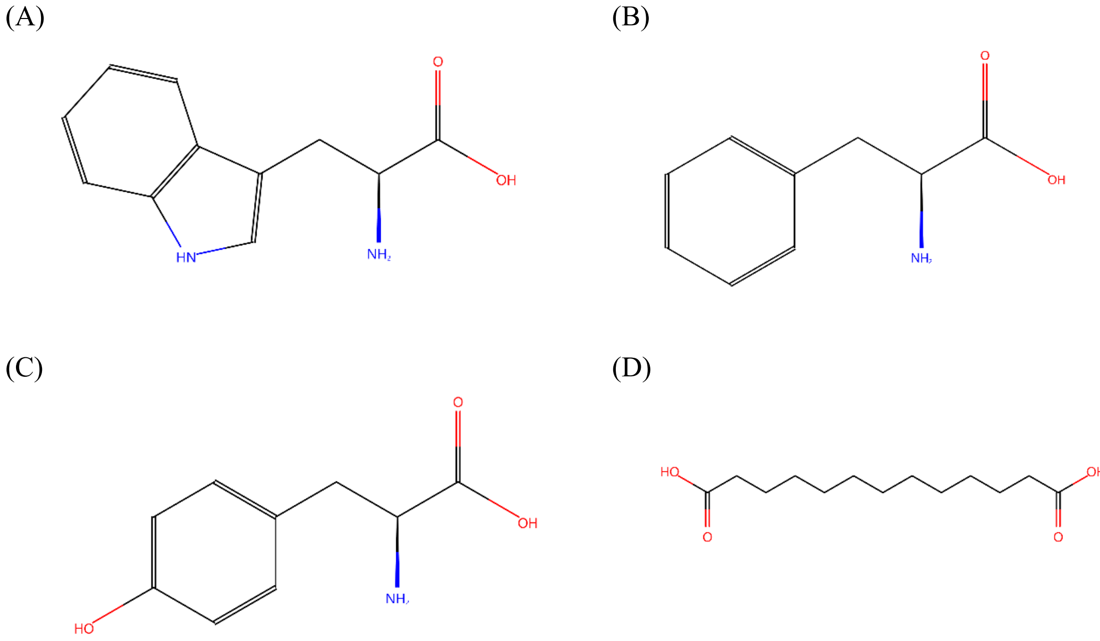

Figure S3. Chemical structures of major metabolites discussed in this study. Aromatic amino acids, including (A): L-tryptophan, (B): L-phenylalanine, and (C): L-tyrosine. (D): Dicarboxylic acids, including 1,11-undecanedicarboxylic acid.

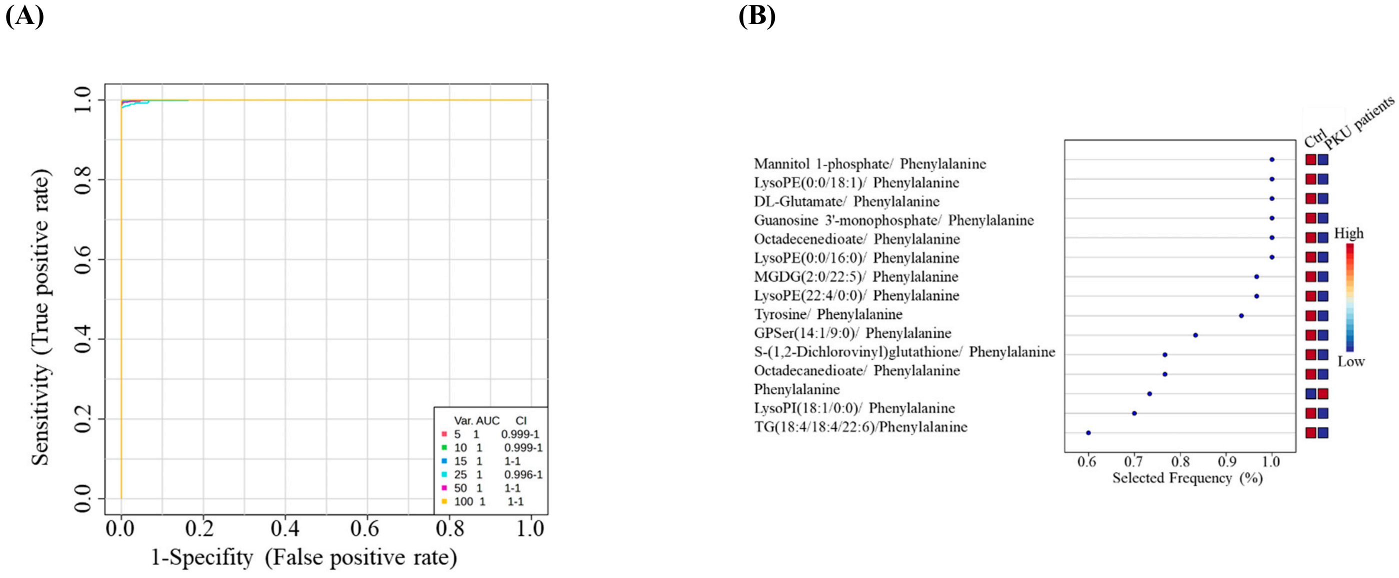

Classification: Restricted

(C)

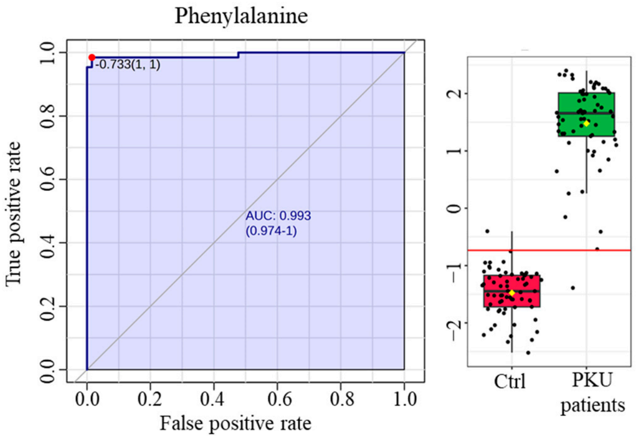

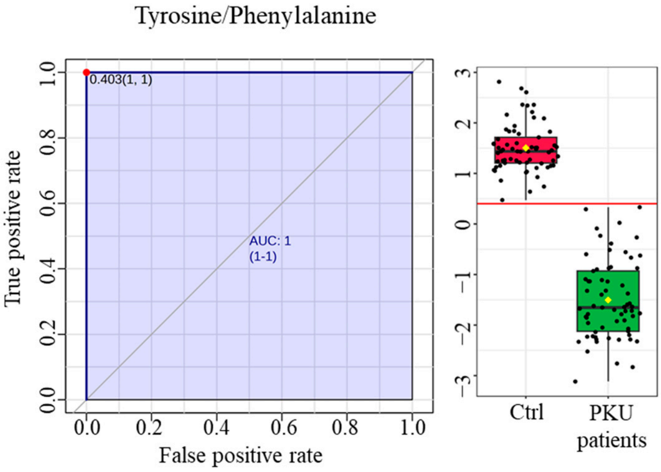

(D)

(E)

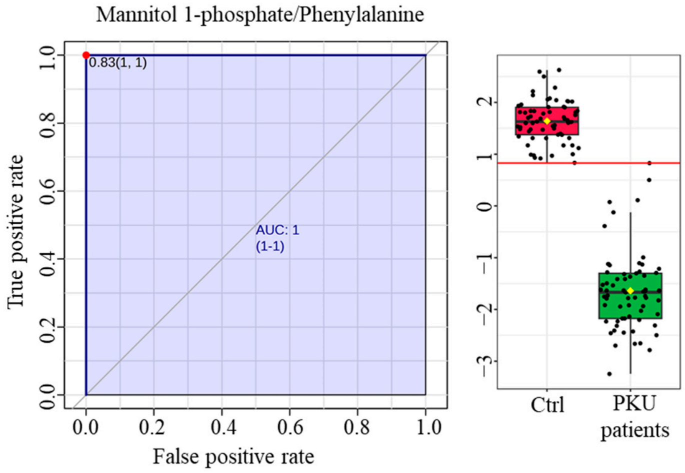

(F)

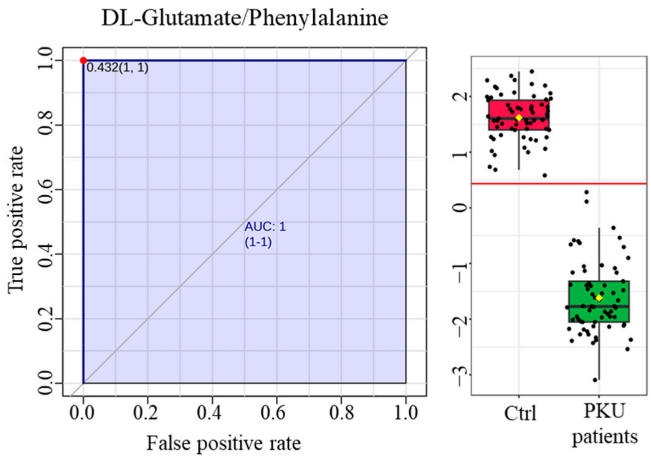

Figure S4 Biomarker evaluation between PKU patients and controls. (**A**) Receiver operating characteristic (ROC) curve comparing PKU patients with controls. It was generated by the OPLS-DA model, with AUC values calculated from the combination of 5, 10, 15, 25, 50 and 100 metabolites. (**B**) A frequency plot shows the significantly dysregulated endogenous metabolites in patients with PKU compared to controls. ROC curves for individual biomarkers: (C) Phen (AUC = 0.993) was upregulated in PKU patients compared with controls. (**D**) Tyr/Phe (AUC= 1), **(E):** Mannitol 1-phosphate/Phe (AUC=1) and **(F)** DL-Glutamate/Phe (AUC=1) were downregulated in PKU patients compared to Ctrl.
